# Fabrication and Characterization of Nanostructured Rock Wool as a Novel Material for Efficient Water-Splitting Application

**DOI:** 10.3390/nano12132169

**Published:** 2022-06-24

**Authors:** Sahar A. El-Gharbawy, Mawaheb Al-Dossari, Mohamed Zayed, Heba A. Saudi, Mohamed Y. Hassaan, Nada Alfryyan, Mohamed Shaban

**Affiliations:** 1 Department of Physics, Faculty of Science, Al-Azhar University (Girls’ Branch), Nasr City, Cairo 11884, Egypt; fouads5649@gmail.com (S.A.E.-G.); heba_saudi@azhar.edu.eg (H.A.S.); myhassaan@yahoo.com (M.Y.H.); 2 Housing and Building National Research Center, 87 El-Tahrir St., Dokki, Giza 1770, Egypt; 3 Department of Physics, Faculty of Science, King Khalid University, Abha 62529, Saudi Arabia; mdosri@kku.edu.sa; 4 Nanophotonics and Applications (NPA) Lab, Department of Physics, Faculty of Science, Beni-Suef University, Beni-Suef 62514, Egypt; m.zayed88ph@yahoo.com; 5 Department of Physics, College of Sciences, Princess Nourah Bint Abdulrahman University, P.O. Box 84428, Riyadh 11671, Saudi Arabia; naalfryyan@pnu.edu.sa; 6 Physics Department, Faculty of Science, Islamic University of Madinah, P.O. Box 170, Al Madinah Al Monawara 42351, Saudi Arabia

**Keywords:** rock wool, nanostructures, water splitting, hydrothermal technique, ball mill

## Abstract

Rock wool (RW) nanostructures of various sizes and morphologies were prepared using a combination of ball-mill and hydrothermal techniques, followed by an annealing process. Different tools were used to explore the morphologies, structures, chemical compositions and optical characteristics of the samples. The effect of initial particle size on the characteristics and photoelectrochemical performance of RW samples generated hydrothermally was investigated. As the starting particle size of ball-milled natural RW rises, the crystallite size of hydrothermally formed samples drops from 70.1 to 31.7 nm. Starting with larger ball-milled particle sizes, the nanoparticles consolidate and seamlessly combine to form a continuous surface with scattered spherical nanopores. Water splitting was used to generate photoelectrochemical hydrogen using the samples as photocatalysts. The number of hydrogen moles and conversion efficiencies were determined using amperometry and voltammetry experiments. When the monochromatic wavelength of light was increased from 307 to 460 nm for the manufactured RW_>0.3_ photocatalyst, the photocurrent density values decreased from 0.25 to 0.20 mA/mg. At 307 nm and +1 V, the value of the incoming photon-to-current efficiency was ~9.77%. Due to the stimulation of the H^+^ ion rate under the temperature impact, the J_ph_ value increased by a factor of 5 when the temperature rose from 40 to 75 °C. As a result of this research, for the first time, a low-cost photoelectrochemical catalytic material is highlighted for effective hydrogen production from water splitting.

## 1. Introduction

In the 21st century, energy is one of the greatest challenges. Nearly 80% of the world’s energy comes from the burning of fossil fuels such as oil, coal and natural gas. Unluckily, these fossil fuels have many drawbacks. Fossil fuels are nonrenewable fuel sources, and within a restricted period, they will inevitably expire. Moreover, the burning of fossil fuels is followed by hazardous CO_2_ emissions owing to the reaction between carbon (C) in fossil fuels and oxygen gas (O_2_). This is the main cause of the reduction in the quantity of oxygen gas in the atmosphere and threatens organisms’ life on earth [1]. The emission of CO_2_ gas into the atmosphere often increases temperatures and causes greenhouse effects and climate change [2]. The challenges for scientists nowadays are to encounter the global energy demand with advances in energy supply and efficiency, along with extenuating the risks of environmental disturbance [3,4]. Solar energy is one of the most promising solutions to address this problem [5], as it is a renewable resource that can be used in photovoltaic cells (solar-to-electricity conversion) and photoelectrochemical (PEC) conversion (solar-to-fuels conversion), as well as water splitting [6].

Hydrogen, on the other hand, is seen as the most promising alternative energy source to nonrenewable fossil fuels [7,8]. In summary, hydrogen-based fuels have received a lot of interest as an alternative energy source to fossil fuels. Hydrogen fuel has the potential to be a cleaner and more sustainable source of energy [9], with a high energy-conversion efficiency and zero carbon emissions [10].

However, since Fujishima and Honda announced the groundbreaking photocatalytic effect of TiO_2_ in 1972 [11], tremendous research has been conducted to generate hydrogen through water splitting using various ways such as electrocatalytic [12,13], photoelectrochemical [14,15,16], and photochemical [17]. Among these, PEC hydrogen production appears to be the most promising effective way to produce hydrogen because it is simple to run, has high efficiency, and does not require additional power to drive the reaction system; it is a low-cost, environmentally friendly, high-performance, and energy-efficient technique [18,19,20,21]. Hydrogen production by solar-water splitting in a PEC cell is a rapidly evolving technology with enormous potential to meet the world’s expanding energy demands [22,23,24,25]. Water splitting in a PEC cell involves two half-reactions: cathodic reduction and anodic oxidation, which is similar to photosynthesis in nature. The photocatalyst absorbs the sun’s light and uses it to trigger chemical processes such as water splitting, which produces H_2_ and O_2_ [26].

Many metal-oxide semiconductors have been developed and used as a photocatalyst for PEC water splitting, such as SnO_2_, ZnO, TiO_2_, SrTiO_3_, Cu_2_O, WO_3_, Fe_2_O_3_, BiVO_4_ and Ta_2_O_5_ [27,28,29,30,31,32,33,34,35]. Furthermore, Pt or Pt-based materials have long been thought to be the most active catalyst for the hydrogen-evolution reaction (HER), but they are expensive and scarce [36,37,38,39,40]. As a result, HER electrocatalysts with low-cost and abundant non-noble elements have been proposed as a possible replacement for Pt-based electrocatalysts. Non-noble metal HER electrocatalysts have better electrocatalytic activity in alkaline electrolytes compared to acidic and neutral electrolytes [41,42]. As a result, transition-metal sulfides (TMS) with an electronic structure comparable to that of noble metals have been investigated as a possible replacement for Pt as an HER electrocatalyst [43,44]. FeS_2_, NiS_2_, CoS_2_, CoSe_2_, CuS and WS_2_ [45,46,47,48,49,50] are some of the TMS that have been widely researched as a replacement for Pt in HER. However, finding an optimal photocatalyst that fits the basic parameters such as an acceptable bandgap for sunlight absorption, suitable band positions for water reduction/oxidation and stability under the requisite reaction conditions with low cost is a hard challenge [51].

Nowadays, scientists seek to explore novel materials (photocatalyst) for hydrogen production-based PEC water splitting. Basalt is the basic material for the creation of rock wool, which belongs to the family of mineral fiber thermal insulators. Because rock wool has a relatively high melting temperature, it can be used as a fire-resistant material [52]. Rock wool waste recycling, on the other hand, is of special relevance in terms of reducing environmental difficulties caused by construction and demolition waste [53]. It was also discovered that including rock wool into lightweight concrete specimens, both with and without heat loading, improved the mechanical properties of the material. RW and glass wool (GW) have been used in town construction [54], ancient city rehabilitation [55], sound insulation [56], industrial buildings [57] and other areas as green building materials.

After thermal loading (at 20, 200, 400 and 600 degrees Celsius), Bahrami and Nematzadeh investigated the mechanical characteristics of pumice lightweight aggregate concrete including rock wool waste (0, 2.5, 5, 7.5 and 10%) [58]. Stonys et al. investigated if mineral wool-production waste might be used to substitute microsilica in the production of refractory concrete [59]. The results demonstrated that integrating rock wool material into the refractory concrete enhanced the specimens’ cold-crushing strength with and without thermal loading. Because of their suitable thermal characteristics, light weight, high tensile strength and low cost, rock wool fibers (RWF) have been widely exploited as insulator materials in recent decades [60]. The RW has not been studied for PEC water splitting.

This research will use a simple and low-cost way to integrate a novel material (RW) as a water-splitting photocatalyst, concentrating on the features and efficiencies of each PEC system. To improve its qualities by increasing surface area, the RW has been transformed to a nanostructured size. A simple and low-cost hydrothermal process was used to create the RW nanomaterials. The influence of particle size (mixed size, 0.03, 0.063, 0.3 and more than 0.3 mm) on RW nanostructure alterations was investigated using XRD, EDAX and SEM. All samples had their optical characteristics tested and were ready to employ in a PEC water-splitting application. For the first time, the effects of the incident monochromatic light wavelength and PEC reaction temperature on the performance of the RW photocatalyst are studied. Moreover, the conversion efficiencies and the produced number of hydrogen moles are obtained.

## 2. Experimental Work

### 2.1. Raw Materials

Fresh rock wool (RW) samples were delivered from Kalabsha kaolinite mine, Aswan, Egypt. The chemical composition of the studied fresh sample is as follows: SiO_2_ (44.9%), CaO (17.8%), Al_2_O_3_ (13.1%), MgO (8.5%), Fe_2_O_3_ (8.8%), Na_2_O (1.9%), K_2_O (1.2%), MnO (0.3%), TiO_2_ (1.9%) and other metal oxide (1.6%). Sodium hydroxide (NaOH, 98.9%) was supplied by ADWIC (Egypt), and hydrochloric acid (HCL, 38%) was supplied by Sigma Aldrich (Germany).

### 2.2. Synthesis of RW Nanostructure

Using a horizontal rolling ball mill devoid of contaminations, the fresh rock wool (RW) was processed for 24 h at 6000 rpm to minimize the sample size. A 1 L cylindrical stainless-steel pot was filled with 80 vol.% of RW particles and monosized 3 mm spherical steel balls. Using a set of sieves, the obtained samples from ball milling were divided into several particle-size samples (Mixed size, 0.03, 0.063, 0.3 and more than 0.3 mm). A total of 4 gm of each sample was dispersed in an aqueous 1 M NaOH solution for 2 h using an ultrasonic stirrer, then transferred to a 250 mL Teflon-lined stainless-steel autoclave and heated at 140 °C for 16 h. The product was recovered by centrifugation and rinsed with 0.1 M HCl after cooling to 27 °C (room temperature). Finally, the RW nanopowder was calcined for 3 h at 700 °C to complete the conversion to the RW crystalline phase. The obtained samples are labeled as RW_mix_, RW_0.03_, RW_0.063_, RW_0.3_ and RW_>0.3_ due to different starting particle sizes; mixed size, 0.03, 0.063, 0.3 and more than 0.3 mm, respectively. 

### 2.3. Samples Characterization

Scanning electron microscopes (SEM; JEOL, JSM-5410LV, Tokyo, Japan) and transmission electron microscopes (TEM, JEOL-2010F, Tokyo, Japan) were used to investigate the surface morphology of manufactured RW nanopowders and to determine the top morphology and interior structure of the samples, respectively. The degree of crystallization and phase composition was determined using a Cu-K X-ray diffractometer (Philips X’Pert Pro MRD, Malvern, UK) with an operating voltage of 40 kV and a scan range of 20° to 80° in 0.05° increments with a count duration of 1 s each time. The chemical compositions were investigated using an energy dispersive X-ray (EDX) spectrometer with a 30 kV accelerating voltage (JEOL JED-2300, Tokyo, Japan). Using a UV-Vis double-beam spectrophotometer, the optical characteristics of the nanopowders were investigated (PerkinElmer, Lambda 950, Boston, MA, USA).

### 2.4. Photoelectrochemical (PEC) Measurements

The PEC analysis was examined by using a workstation (Orga-Flex, Paris, France) in the two-electrode system; Pt and graphite sheets were the two electrodes used. A total of 0.05 g of Rw sample was dispersed in 0.3 M Na_2_S_2_O_3_ aqueous solution as electrolyte medium (pH = 7.0) that consumes the photogenerated holes from the photocatalytic surface. The photocurrent was recorded under irradiation by a 400-Watt Xenon lamp (Newport, UK) as the simulative solar light with applied voltage (V) ranged from 0 to +1 V.

## 3. Results and Discussion

### 3.1. XRD Structure Analysis

**Figure 1:**
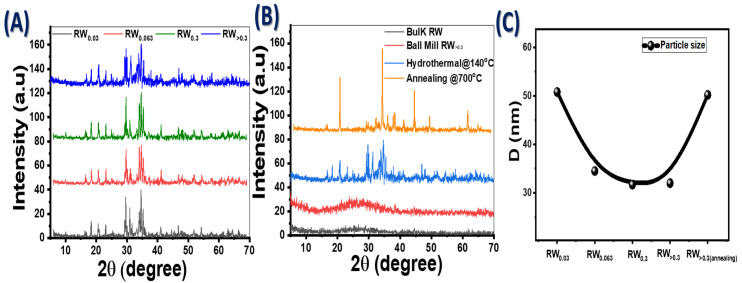
(**A**) Standard XRD patterns for fabricated nanostructure RW at different particle sizes, (**B**) XRD patterns of bulk RW and RW_>0.3_ after ball milling, hydrothermal technique and annealing; and (**C**) particle size for all RW samples.

XRD spectra for fabricated nanostructure RW were obtained to analyze the crystal structures and phase purity (Figure 1A). For RW_0.03_, there are three peaks of Sr_11_Mg_2_Si_10_, located at 2θ = 29.59°, 30.91° and 34.06° with Miller indices (−511), (−405) and (114) according to 01-086-2488 JCPDS card. There are two main peaks of Ca_2_Fe_15.6_O_25_ and K_2_S located at 2θ = 34.54° and 35.28° with Miller indices (200) and (020) according to 01-078-1184 and 01-071-3427 JCPDS cards, respectively.

For RW_0.063_, there are two peaks of Sr_11_Mg_2_Si_10_, located at 2θ = 29.71 and 34.05° with Miller indices (312) and (114) according to 01-086-2488 JCPDS card. There are three additional main peaks of CaSO_4_, Ca_2_Fe_15.6_O_25_ and K_2_S located at 2θ = 23.15°, 34.68° and 35.31° with Miller indices (101), (200) and (013) according to 00-026-0328, 01-078-1184 and 01-071-3427 JCPDS cards, respectively. For RW_0.3_, there are two peaks of Sr_11_Mg_2_Si_10_, located at 2θ = 29.70° and 34.11° with Miller indices (312) and (114) according to 01-086-2488 JCPDS card. There are three other main peaks of Ca_2_Fe_15.6_O_25_, K_2_S and AlH_4_O_6_P located at 2θ = 34.65°, 35.32° and 39.95° with Miller indices (200), (013) and (114) according to 01-078-1184, 01-071-3427, and 01-070-0310 JCPDS cards, respectively. The observed peaks for RW_>0.3_ are similar to the observed peaks for RW_0.3_. After annealing of the sample RW_>0.3_, Figure 1B, there are two high peaks of Ca_2_Fe_22_O_33_ located at 2θ = 20.81° and 34.32° with Miller indices (018) and (021) according to 01-077-0565 JCPDS card, and three other peaks due to Fe_3_O_12_P_3_Sr located at 2θ = 38.32°, 44.50° and 61.54° with Miller indices (312), (123) and (471) according to the card 01-089-8396. There are other minor peaks for all samples due to the natural chemical composition of RW. As shown in Figure 1B, the bulk RW exhibited an amorphous structure. The crystallinity of the hydrothermally prepared RW sample improved after annealing at 700 °C.

The angle of X-ray diffraction is inversely related to the interplanar distance according to Bragg’s equation: [61,62,63,64]

(1) m λ=2 d sinθ

where λ, d, m and θ are the incident X-ray wavelength, the interplanar distance, the order of diffraction and the Bragg’s diffraction angle, respectively. The crystallite size (D) of the ZnO films was estimated from the Debye–Scherrer formula [65,66]:

D = 0.94 λ/β cosθ(2)

where λ = 0.154 nm and (β) is the full width at half maximum intensity. The crystallite sizes for RW_0.03_ and RW_0.3_ are nearly 50.8 and 31.7 nm, respectively. After annealing, the crystallite size is increased for RW_>0.3_ from ~32.0 to 50.2 nm as shown in Figure 1C, i.e., the crystallite size of the hydrothermally generated samples decreases as the initial particle size of ball-milled natural RW increases. 

The texture coefficient (T_C_) represents the texture of a particular plane, the deviation of which from unity implies preferred growth. This factor is calculated using the following relation [67]:

(3) $$T_{C}\left(\mathrm{hkl}\right)=\frac{I\left(\mathrm{hkl}\right)/I_{o}\left(\mathrm{hkl}\right)}{N^{-1}\Sigma_{n}I\left(\mathrm{hkl}\right)/I_{o}\left(\mathrm{hkl}\right)}$$

where I(hkl) and I_o_(hkl) are the measured relative intensity of a plane (hkl) and the standard intensity of the plane (hkl) taken from the JCPDS data; N is the reflection number; and n is the number of diffraction peaks. T_C_s values were calculated for the highest five main peaks detected in the XRD patterns. The preferred orientation for RW_0.03_, RW_0.063_, RW_0.3_ and RW_>0.3_ nanopowders was (200), (200), (200) and (021) with values 2.36, 1.13, 1.07, 1.06 and 1.17, respectively.

### 3.2. Chemical Composition

**Figure 2:**
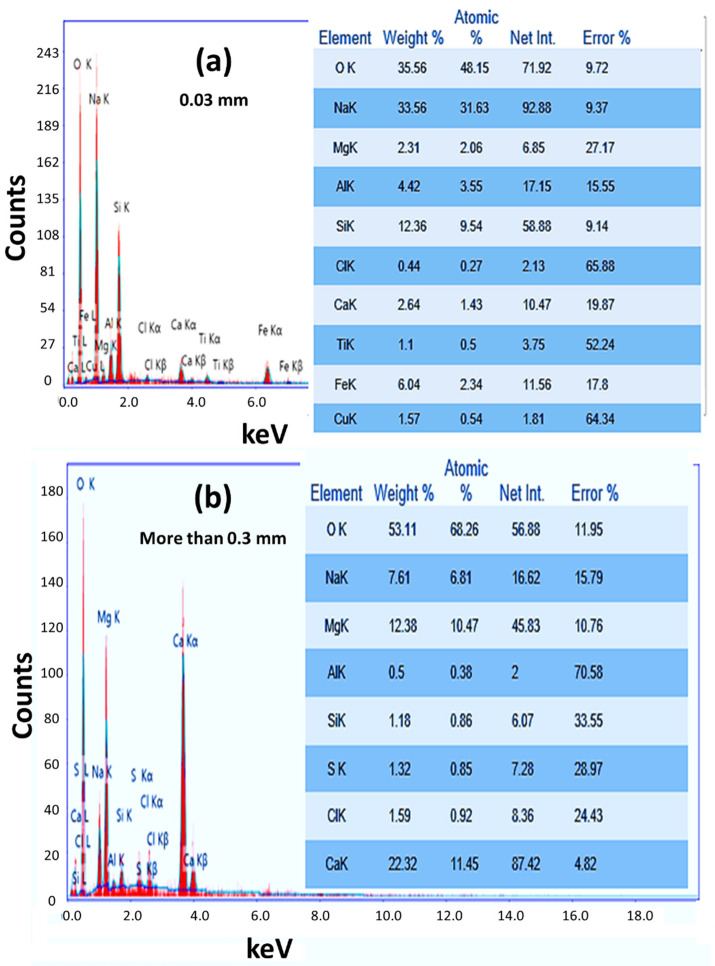
EDX patterns for nanostructured (**a**) RW_0.03_ and (**b**) RW_>0.3_.

Figure 2 shows the EDX patterns for nanostructured RW_0.03_ and RW_>0.3_. The quantitative chemical compositions for these samples are presented in the inset tables of Figure 2a,b. The main signals for RW_0.03_ are O (35.56 wt.%), Na (33.56 wt.%), Si (12.36 wt.%), Fe (6.04 wt.%), Al (4.42 wt.%), Ca (2.64 wt.%), Mg (2.31 wt.%), Cu (1.57 wt.%) and Ti (1.1 wt.%). 

This indicates the existence of SiO_2_, Na_2_O, Fe_2_O_3_, Al_2_O_3_, CaO, MgO, CuO and TiO_2,_ as confirmed by the XRF analysis. The high signal of Na may come from the RW and the used materials and solutions during the hydrothermal technique and washing process. The Na and Cl signals resulted from the formation of NaCl because of the reaction of the used 1 M NaOH during the hydrothermal technique and 0.1 HCl that was used during the washing process according to the exothermic reaction HCl (aq) + NaOH (aq) → NaCl (aq) + H_2_O + *heat*. The Sample RW_>0.3_ shows higher signals for O, Ca, Mg and Cl, whereas the signals of Na, Si and Al are lower than that of RW_0.03_. In addition, the signals of Fe, Ti and Cu disappear. The existence of S (1.32 wt.%) and the increase in oxygen signal indicate the formation of SO_3_. Similar signals were reported for RW by many authors [68,69,70]. 

### 3.3. Surface Morphology 

**Figure 3:**
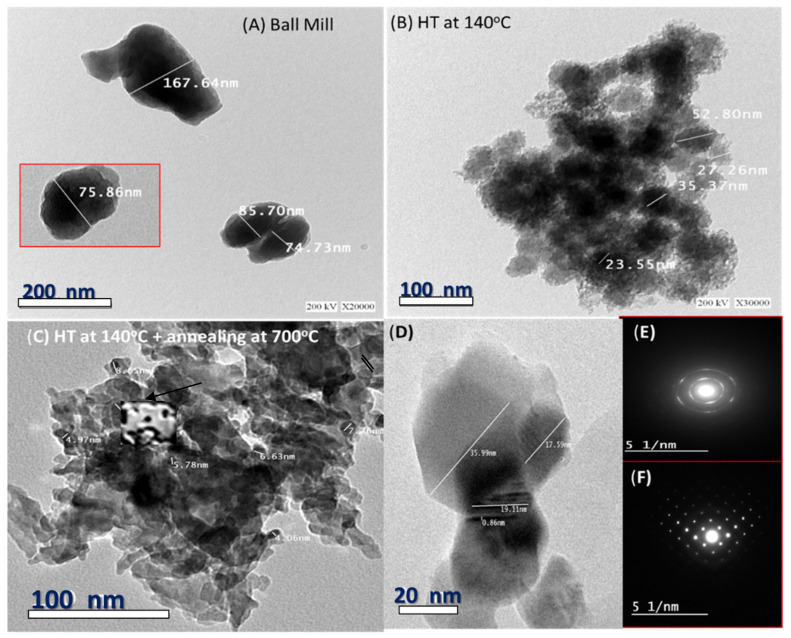
HR-TEM of the RW_0.03_ after (**A**) ball mill, (**B**) hydrothermal technique at 140 °C, and (**C**,**D**) annealing at 700 °C; (**E**,**F**) selective-area electron diffraction (SAED) after annealing.

Figure 3A–D illustrates high-resolution transmission electron microscopy (HR-TEM) micrographs for nano-rock-wool powder after ball milling, hydrothermal technique at 140 °C and after annealing at 700 °C. The micrograph after ball milling (Figure 3A) shows nonspherical nanoparticles with diameters ranging from ~75 nm to ~168 nm. Figure 3B reveals networks from very fine sphere nanoparticles, which self-assemble to form semispherical spongy surfaces with diameters ranging from ~23 to 53 nm. After annealing, in Figure 3C,D, these particles are arranged as a network of concentric spheres in an elongated manner attached in a preferred orientation. The size of the self-assembly pores is less than 9 nm. Besides the spherical shapes, hexagonal shapes are also detected, as shown in Figure 3D. Figure 3D also indicates that the sample after annealing at 700 °C shows a smaller particle size than before. Many authors previously reported that the change in primary average particle size as a function of temperature shows two decoupled zones: particle shrinkage due to densification up to a particular temperature between 700 °C and 900 °C, and particle growth due to coarsening behavior above this temperature [71,72,73]. Because the surfaces of the particles in Figure 3B form semispherical spongy surfaces, then the annealing of our sample lowers its particle size owing to the densification impact being greater than coarsening behavior. Note that the melting point of rockwool is 1177 °C, which is higher than the used annealing temperature in this study. In addition, after annealing, the sample crystallinity is improved, as also confirmed by the ring and spot selective-area electron diffraction (SAED) in Figure 3E,F. This sample shows bright spots and rings and thereby a polycrystalline nature, as confirmed by the XRD. The obtained interplanar distance is 0.86 ± 0.1 nm. Figure S1 (Supplementary Data) shows HR-TEM images of the (A) RW_0.063_ sample and (B–D) RW_>0.3_ sample after hydrothermal technique at 140 °C and annealing. All images show particles in the nanoscale. In some regimes, these particles are self-assembled to form nanoporous features. The inset of Figure S1D shows the ring SAED pattern of RW_>0.3_, which confirms the polycrystalline nature of the sample as confirmed by the X-ray analysis (Figure 2).

**Figure 4:**
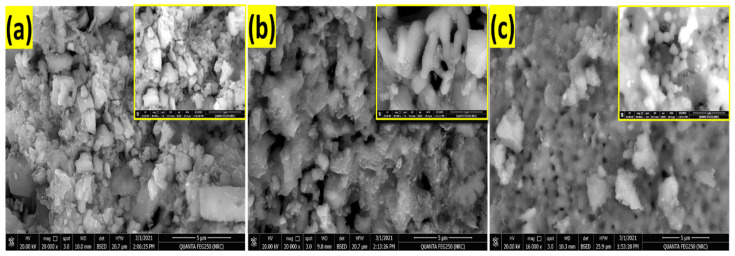
SEM images of top view for RW nanostructured; (**a**) RW_mix_, (**b**) RW_0.03_ and (**c**) RW_>0.3_. The insets show magnified SEM images.

Figure 4 illustrates SEM images of the prepared RW_mix_, RW_0.03_ and RW_>0.3_ samples. The sample of RW_mix_ (Figure 4a) displays mixed morphologies with random distributions with sizes ranging from nano to micro. The microsize particles result from the coalescence of small nanoparticles. The surface of this sample is the roughest. For the RW_0.03_ sample, (Figure 4b) the surface roughness is decreased and the nanoparticles coalesce and self-assemble to form agglomerations of particles and rods with pores of diameters in the range of 603 ± 303 nm. For the RW_>0.3_ sample (Figure 4c) the coalesced nanoparticles are smoothly assembled to form a continuous surface with distributed spherical nanopores on the surface. The average diameter of the agglomerated particles is 662 ± 250 nm, whereas the pore diameter is in the range of 440 ± 116 nm.

### 3.4. Optical Analysis

**Figure 5:**
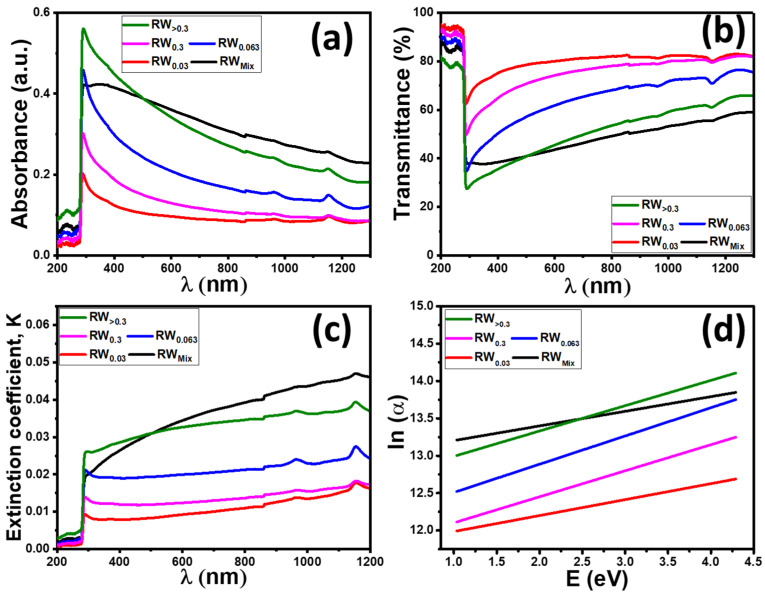
Optical spectra of RW nanostructured (**a**) absorbance, (**b**) transmittance, (**c**) ln(α)-E to determine Urbach energy (E_U_); and (**d**) the extinction coefficient for the fabricated RW as a function of the wavelength.

UV-Vis. transmission/absorption spectroscopy is a very important technique to analyze the optical properties (A%, T%) and direct bandgap of semiconductor nanomaterials. Figure 5a,b shows the absorbance (A%) and transmittance (T%) spectra of RW samples fabricated at different particle sizes. The RW_>0.3_ sample shows sharp absorption bands in the UV region (below λ = 400 nm) corresponding to electron valence band/conduction band transitions. The left edge of the absorption band is slowly shifted to a lower wavelength as the particle size decreases, indicating the increase in the optical bandgap. In addition, it is easy to notice that absorbance is slightly decreased with decreasing particle size to RW_0.03_.

The area under the absorption curve in UV-Vis ranging from 200 to 500 nm is highly essential to indicate the transient light response of the photoanode; the RW_>0.3_ has the highest area under the curve, indicating a greater number of absorbed photons and enhancing PEC application. The transmission spectra show that the films are highly transparent in the visible region, about 82%. The optical transmission of samples is slightly decreased with increasing particle size due to the increase of the absorption. The highest transmittance for RW_>0.3_ is also related to surface morphology, in which the RW_>0.3_ shows the formation of uniform nanoporous and smooth top surfaces according to SEM images in Figure 4c.

Using the obtained data for absorbance (A), the absorption coefficient (α) for RW samples with different particle sizes was calculated by using the following equation [74,75]:

(4) $$\alpha =2.303\frac{A\rho }{\mathrm{LC}}$$

where ρ is the density of nanopowder, L is the length of the quartz cell (=1.0 cm) and C is the concentration of the nanopowder in the suspension.

The extinction coefficient (K) can be calculated from obtained (α) values according to the following equation:

(5) $$K=\frac{\alpha \lambda }{4\pi }$$

The extinction coefficient represents the ability of the sample to absorb the electromagnetic waves due to inelastic scattering actions. Figure 5c shows that the k values of the RW samples depend on the wavelength. The K values at 287 nm and 500 nm are listed in Table 1. The k values for RW_0.03_, RW_0.063_ and RW_>0.3_ are 0.009, 0.02 and 0.025, respectively. These values indicate that the k value is sharply increased with an increasing particle size of natural RW in the UV region (λ = 287 nm). In addition, the same behavior is seen in the visible light region (λ = 500 nm), in which the k values for RW_0.03_, RW_0.063_ and RW_>0.3_ are 0.008, 0.019 and 0.03, respectively as shown in Table 1. RW_>0.3_.

The absorption coefficient usually shows exponential energy dependence near the fundamental absorption edge (Urbach tail) according to the following equation [76]:

(6) $$\alpha =\alpha_{0}e^{\left(\frac{h\nu }{E_{u}}\right)}\;$$

where α_0_ is a constant and E_U_ is the Urbach energy. The Urbach tail value (E_U_) determines the width of the tail in the valence and conduction bands, which appears due to the disorder in the material. It is ascribed to the disorder in the material that indicates the tail in the valence and conduction bands [77]. Figure 5d shows the plots of lnα versus hυ, and the values of the E_U_ were obtained from the slopes of the linear fitting of this Figure. The obtained values of the E_U_ are listed in Table 1. E_U_ increases from 213 to 358 meV as the particle size of natural RW increases from RW_0.03_ to RW_>0.3_ and then decreases to 195 meV with the RW_mix_. The highest value for E_U_, 358 meV, obtained for RW_>0.3_, may be attributed to the disorder nature and voids that appear on the film surface as shown from the SEM image (Figure 4c).

**Figure 6:**
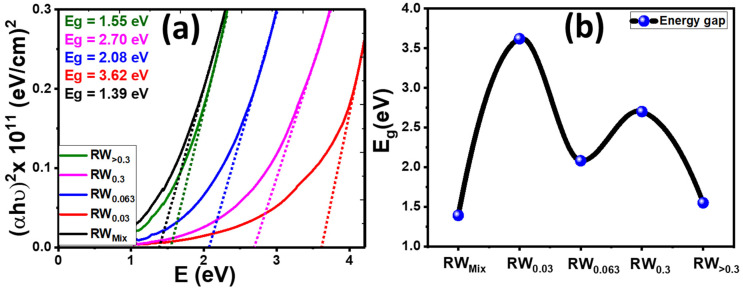
(**a**) (αhν)^2^ versus hν and (**b**) variation of E_g_ for all RW samples.

The direct optical band gap of nanopowders was calculated by using the Tauc equation [78]:

(7) $${\left(\alpha h\upsilon \right)}^{2}=G\;\left(h\upsilon \;-E_{g}\right)$$

where G is a constant, υ is the frequency of the photon, h is Planck’s constant, E_g_ is the direct bandgap between the conduction (C.B) and valence band (V.B) and α is the absorption coefficient. If plotted ${\left(\alpha h\upsilon \right)}^{2}$ versus hν and a tangent line is drawn from the intercept point on the curve, the intersection of the tangent line with the horizontal axis (hυ axis), Figure 6a indicates the bandgap transition ($E_{g}=h\upsilon$ when α=0).

The obtained Eg values are plotted in Figure 6b for all RW samples and listed in Table 1. The estimated energy band gap of the RW_Mix_, RW_0.03_, RW_0.063_, RW_0.3_ and RW_>0.3_ nanopowders are 1.39, 3.62, 2.08, 2.7 and 1.55 eV, respectively. The Eg of nanostructured RW is blue-shifted from 3.62 to 1.55 eV as the particle size of natural RW increased from RW_0.03_ to RW_>0.3_. The blue shift may be related to the development of a resonance structure (state) in the density of states and the split-off band by introducing deep states into the bandgap [79]. From Figure 1B, Figure 4c and Figure 6b, the RW_>0.3_ nanopowder is the most suitable for application in water splitting. The sample of RW_0.063_ does not match the tendency with others, this may be ascribed to the lower crystallite size (34.5 nm) and morphology changes as shown by the provided HR-TEM image in Figure S1A (Supplementary Data). The inset image shows the existence of the nanoporous features due to the self-assembly of the high density of the nanoparticles. The self-assembly of nanoporous surfaces improves optical absorption and reduces the bandgap. In general, for particles with diameters greater than 10 nm, the bandgap reduces as the crystallite size decreases. Due to the quantum size effect, the verse appears when the crystallite size is within 1–10 nm, i.e., due to the Moss–Bustein effect, as the particle size decreases the optical band gap can be widened by shifting towards the higher frequency side gradually [80]. In addition, as the size of the nanoparticles decreased, the surface defect states reduce the bandgap more than the bulk counterpart [81].

### 3.5. Photoelectrochemical (PEC) Performance of the Samples

The photoelectrochemical (PEC) water-splitting behavior of the RW samples was measured using an Orga-Flex workstation with two-electrode configuration under light irradiation of a 400 W Newport Xenon lamp. The illumination power was adjusted at the electrode to be 100 mW/cm^2^. As a redox electrolyte for solar water splitting, 5 mg of each RW powder sample was disseminated in a 0.3 M Na_2_SO_4_ (pH = 7) aqueous solution. At room temperature (25 °C), linear sweep voltammetry is performed in the range of 0 V to 1.0 V.

**Figure 7:**
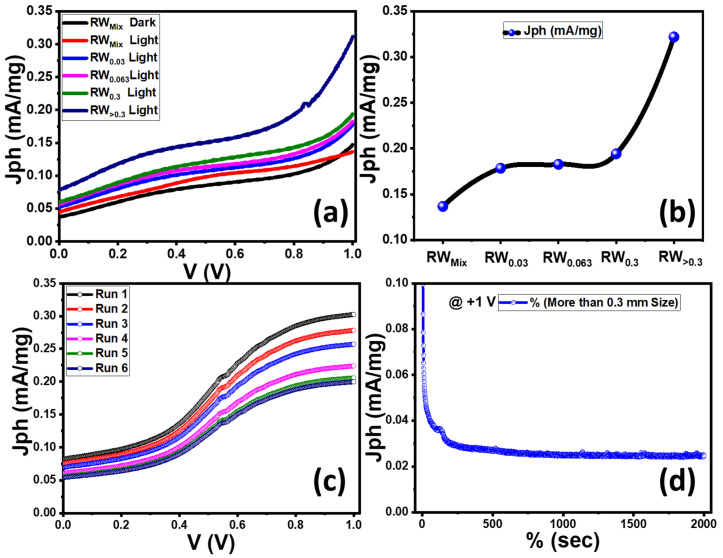
(**a**) Photocurrent density versus the applied voltage (J_ph_–V) for all RW samples; (**b**) variation of J_ph_ @1 V for RW samples of different sizes; (**c**) reproducible study of J_ph_–V curves for RW_>0.3_; and (**d**) J_ph_–Time Stability for RW_>0.3_.

To clarify the effect of particle size of the RW photocatalyst, PEC characterizations in a two-electrode system were studied in depth. From Figure 7a, the RW_mix_ sample has a very small current density (J_ph_) in dark, which reaches a maximum value of 0.05 mA/mg@1 V. This value increased to 0.15 mA/mg under light irradiation at an applied external voltage of 1 V. For individual particle size samples, with increasing particle size, the photocurrent density increased (J_ph_) as shown in Figure 7a,b to reach 0.32 mA/mg at 1 V for the RW_>0.3_ sample. This value is near twice the J_ph_ value of the RW_Mix_ sample. Furthermore, the onset potential of the RW_>0.3_ sample shifts substantially positively, indicating that the charge separation and transfer efficiency of RW has greatly increased [82,83,84,85]. The huge number of active sites generated by unsaturated coordination from the ultrathin nanoporous RW structure as demonstrated in the SEM images might account for these results. This sample (RW_>0.3_) also has the highest photocurrent density because it has the largest area under the absorption curve at wavelengths between 250 and 500 nm, indicating that it has a considerable number of absorbed photons in the UV-Vis range. Surface nanostructures, according to Ojha et al., improve the well-defined interfaces between the catalyst and the electrolyte, enable quick charge transfer, and create highly open designs that increase the number of surface atoms, all of which lead to increased electrochemical performance [86]. In addition, well-ordered crystalline states limit the transmission of visible light, improve absorption, and form stable highly porous structures [87]. Moreover, the fall in oxygen percentage seen by reducing the beginning particle size, as illustrated in Figure 2, can be attributed to the size reduction creating oxygen vacancies at the surfaces of the generated nanostructures. This is similarly connected to the decrease in crystallinity found in samples with a starting particle size of less than 0.3, as illustrated in Figure 1A. Defects such as oxygen vacancies impair carrier separation efficiency, and as a result, sample performance, which might explain why the RW_>0.3_ had the highest J_ph_ values [88].

The effective reproduction of H_2_ using photocatalysts is very important concerning economic issues. The (J_ph_–V) curves of the RW_>0.3_ sample were measured under the illumination of a 400 W Xe-lamp several times to study the reproducibility of the RW catalyst, as shown in Figure 7c. There is a small change in the value of the J_ph_ after carrying out the measurements six times. The value of the J_ph_ is changed from 0.32 to 0.22 mA/mg @1 V. On the other hand, the stability test is a very important parameter for PEC in practical applications. The stability of the fabricated RW electrode during the PEC water-splitting method was examined by measuring the change in J_ph_ for an elongated time. Figure 7d illustrates a graph of J_ph_ versus time measured at an applied voltage of 1 V for 2000 s. The measurements were carried out in 0.3 M Na_2_S_2_O_3_ electrolyte solution under light irradiation of 100 mW/cm^2^ from a 400-W Xenon lamp. The slopes of this curve reflect the stability of the photoelectrodes; a smaller slope indicates better stability. During these experiments, the J_ph_ values were decreased in the first period due to the minimal photochemical corrosion process [89]. Above 50 s, the J_ph_ values remained constant at nearly 0.025 mA/mg due to the increase in the accumulation of the ionic charges. This result suggests that the photoanode has suitable chemical stability and a long lifetime to work in the H_2_-production cell.

**Figure 8:**
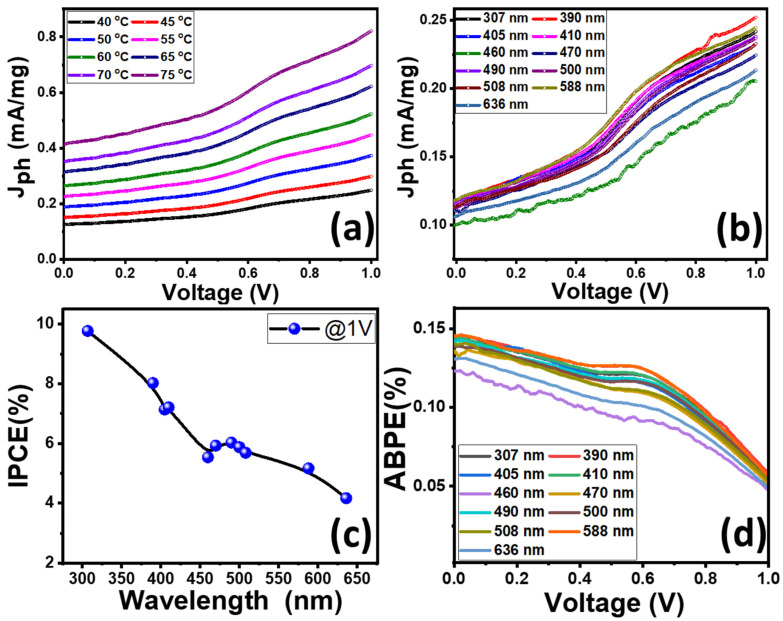
(**a**) Effect of (**a**) temperature and (**b**) wavelength of the monochromatic illumination on the J_ph_–V curves; conversion efficiencies (**c**) IPCE% versus the monochromatic wavelength and (**d**) ABPE% versus the applied bias at different wavelengths for RW_>0.3_ sample.

The influence of temperature from 25 to 60 °C, as well as the wavelength of the illuminating monochromatic light from 307 to 636 nm, as shown in Figure 8a,b, were investigated. The effect of temperature on the J_ph_ value for the photoelectrochemical H_2_O water-splitting reaction is shown in Figure 8a. By increasing the temperature from 40 to 75 °C, the J_ph_ value is increased to reach 0.82 mA/mg. Temperature appears to play a substantial effect in enhancing photocurrent densities, according to these findings. This is due to the temperature influence increasing the incentive of the H^+^ ion rate [90].

The effect of monochromatic-wavelength light from 307 to 636 nm on the J_ph_ value of RW_>0.3_ is shown in Figure 8b. From the figure, the nonlinear behavior of the J_ph_–V curve is obtained under different monochromatic light at an applied voltage of 1 V. The photocurrent density values increased to reach their maximum value (0.25 mA/mg) at 390 nm, then the value decreased to reach 0.21 mA/mg at 636 nm. The minimum value (0.20 mA/mg) was reported at 460 nm. This variation with the wavelength indicates the ability of the prepared RW_>0.3_ photocatalyst to work in a wide wavelength range. These J_ph_ values match well with the optical analysis in Figure 5.

To understand in detail the improved PEC performances of the fabricated RW_>0.3_, incident photon-to-charge efficiency (IPCE) was estimated under monochromatic illumination conditions, as shown in Figure 8c. The IPCE (external quantum efficiency) is a measure of the ratio of the number of photogenerated electrons used in the redox reactions to the number of incident monochromatic photons as a function of the wavelength. A higher value of IPCE indicates the improved production of photoexcited charge carriers. The IPCE was calculated at an applied potential of +1 V from Equation (8) [91]:

$$\mathrm{IPCE}=\frac{\;\mathrm{Total}\;\mathrm{energy}\;\mathrm{of}\;\mathrm{converted}\;\mathrm{electrons}}{\mathrm{Total}\;\mathrm{energy}\;\mathrm{of}\;\mathrm{incident}\;\mathrm{photons}}$$

(8) $$=\frac{J_{\mathrm{ph}}\left(\mathrm{mA}/\mathrm{mg}\right)}{P_{\mathrm{light}}\left(\mathrm{mw}/\mathrm{mg}\right)}\;\frac{1240}{\lambda \;\left(\mathrm{nm}\right)}\times 100\;\left(\%\right)$$

The IPCE was estimated at an applied potential of 1 V, J_ph_ (mA/mg) is taken at wavelengths ranging from 307 to 636 nm of the incident light; λ is the wavelength of the illuminating monochromatic photon and P_light_ is the illuminating light power density. The value of IPCE for RW_>0.3_ increases with decreasing the wavelength of the incident photon. RW_>0.3_ shows strong photoactivity in the UV-Visible light region and enhancement of IPCE throughout the wavelength range of 307–460 nm with an IPCE value of ~9.779% at 307 nm as observed in Figure 8c.

To further clarify the performance of the RW, we calculated the applied bias photon-to-current efficiency (ABPE), as shown in Figure 8d. ABPE can provide analytical measurements that characterize the development of photocatalytic performance concerning the applied external potential. The ABPE was calculated using Equation (9) [92]:

(9) $$\mathrm{ABPE}=J_{\mathrm{ph}}\left(\mathrm{mA}/\mathrm{mg}\right)\;\frac{\left(1.23-E_{\mathrm{applied}}\right)}{P_{\mathrm{light}}\left(\mathrm{mA}/\mathrm{mg}\right)}\times 100\;\left(\%\right)$$

As seen in Figure 8d, the maximum value of ABPE efficiency for RW_>0.3_ achieved ~0.15% at a wavelength of 390 nm. A peak value of ~0.13% is observed at 0.56 V for 390 nm. The peak value slightly shifts to a higher voltage by increasing the wavelength. Both IPCE and ABPE have a maximum value at a wavelength range < 460 nm. As a result, the observed improved PEC performance could be attributed to the number of photons absorbed as illustrated in the optical properties section, which leads to an increase in the generation of charge.

**Figure 9:**
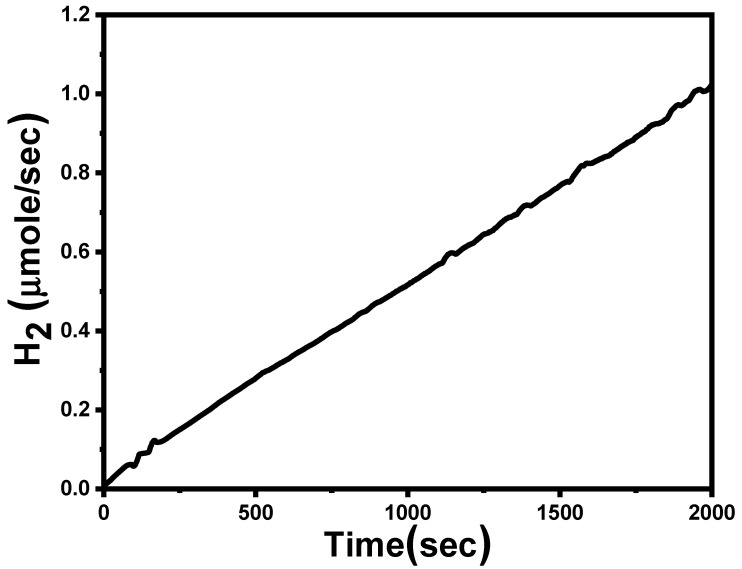
The number of H_2_ moles produced as a function of time.

The number of produced hydrogen moles can be calculated from Faraday’s law of electrolysis [93]:

(10) H2(moles)=2 ∫Jph dtF

where *F* is the Faraday constant (96500 C/mol), J_ph_ is the current density in A/mg and t is the time in sec. Based on the amperometric J_ph_–t curve, the calculated number of generated *H*_2_ moles per active area is 1040.423 μmol/h.mg for RW_>0.3_, as shown in Figure 9. The rapid growth in the amount of H_2_ indicated the excellent stability of the RW_>0.3_ photoelectrodes.

In addition, Table 2 shows a comparison between our optimized RW nanocatalyst and recently reported nanotextured catalysts [94,95,96,97,98,99,100,101,102,103,104]. The comparison is carried out in terms of photocatalyst composition, electrolyte, light power or source and catalytic performance parameters (H_2_ moles, J_ph_ or IPCE% values).

## 4. Conclusions

Rock Wool (RW) nanostructures of various sizes and morphologies were created using a combination of ball mill and hydrothermal methods, followed by annealing. Different methodologies were used to explore the morphologies, structures, chemical compositions and optical characteristics. The effect of initial particle size on the characteristics and photoelectrochemical performance of RW samples generated hydrothermally was investigated. As the starting particle size of ball-milled natural RW rises, the crystallite size of hydrothermally formed samples drops from 70.1 to 31.7 nm. Starting with larger ball-milled particle sizes, the nanoparticles consolidate and seamlessly combine to form a continuous surface with scattered spherical nanopores. The samples are used to generate photoelectrochemical hydrogen by splitting water. The number of hydrogen moles and conversion efficiencies were calculated using amperometry and voltammetry measurements. The photocurrent density values for the manufactured RW_>0.3_ photocatalyst electrode decreased from 0.25 to 0.20 mA/mg as the monochromatic-wavelength light increased from 307 to 460 nm. At 307 nm and +1 V, incident photon-to-current efficiency was ~9.77%. The J_ph_ value increases by 5 times when the temperature rises from 40 to 75 °C due to the temperature influence on the H^+^ ion rate. The calculated number of generated H_2_ moles per active area is 1040.423 μmol/h·mg. The current study highlights a low-cost nanostructured RW as a photoelectrode material that may be further enhanced and employed for successful hydrogen production. Finally, we will concentrate our future efforts on electrochemical characterization and corrosion investigations of our optimized nanocatalyst, as well as the incorporation of plasmonic nanoparticles to improve the photocatalyst’s stability and performance.

## Figures and Tables

**Table 1:** The calculated parameters for RW as crystallite size (D), texture coefficient (T_C_), energy gap (E_g_), Urbach energy (E_U_) and extinction coefficient (K).

Samples	D (nm)	T_C_		E_g_ (eV)	E_U_ (meV)	K	
		(hkl)	Value			λ = 287 nm	λ = 500 nm
**RW_Mix_**	70.1	(200)	2.36	1.39	195.75	0.018	0.029
**RW_0.03_**	50.8	(200)	1.13	3.62	213.47	0.009	0.008
**RW_0.063_**	34.5	(200)	1.07	2.08	377.69	0.02	0.019
**RW_0.3_**	31.7	(200)	1.06	2.7	348.18	0.013	0.011
**RW_>0.3_**	50.2	(021)	1.17	1.55	358.72	0.025	0.03

**Table 2:** Comparison between the present photocatalyst and the recently reported literature in terms of composition, electrolyte, light power or source, main performance indicators (H_2_ moles, J_ph_, or IPCE% values).

Photocatalyst	Electrolyte	Light Power	Performance	Ref.
Pt-loaded yolk–shell TiO2@SiO_2_ nanoreactors (50 mg)	80 mL mixture of methanol (40 mL) and water (40 mL)	300 mW/cm^–2^ (Simulated sunlight)	H_2_ moles = 24.56 mmol·g^−1^·h^−1^	[94]
ZnO (5 mg) La/ZnO (5 mg) La/ZnO/CNTs (5 mg)	80 mL aqueous solution containing 10% of glycerol	300 W Xe light source	H_2_ moles = 10.2 mmol/h H_2_ moles = 145.9 mmol/h H_2_ moles = 184.8 mmol/h	[95]
V and La co-doped ZnO/CNTs nanocomposite (10 mg)	100 mL water and methanol	300 W Xe lamp	H_2_ moles = 267 μmol·h^−1^·g^−1^	[96]
Ultra-fine Cu (6 wt%) decorated hydrangea-like TiO_2_ (20 mg)	100 mL 10 vol% aqueous solution methanol	300 W Xe lamp	H_2_ moles = 3.7 mmol·h^−1^·g^−1^	[97]
Hierarchical porous NiO anchored on graphitic carbon nitride with nitrogen vacancies	10 mL sacrificial reagent triethanolamine and 90 mL H_2_O	420-nm (3 W) LED light illumination	H_2_ moles = 170.60 μmol·g^−1^·h^−1^	[98]
Hierarchical e 0.75% SiO_2_@ZnIn2S4 marigold flower like nano heterostructure (0.5 g)	700 mL 0.5 M aqueous KOH and purged with Argon for 30 min	-	H_2_ moles = 6730 μmol/h·g	[99]
-Fe_2_TiO_5_/ZnO Nanodendrite Heterojunction Array -Co-Pi/Fe_2_TiO_5_/ZnO ND heterojunction array	0.3 M Na_2_SO_4_ in K_3_PO_4_ buffer solution at pH 7.5	500 W xenon lamp	J_ph_ = 1.04 mA cm^–2^ at 1.23 V vs. RHE J_ph_ = 2.14 mA cm^–2^ at 1.23 V vs. RHE	[100]
Cu/CuO Nanoporous photoelectrode	Sewage water	400 W Newport Xenon lamp	IPCE = 14.6% J_ph_ = 4.7 mA·cm^−2^	[101]
Au/Poly M-Toluidine	Na_2_S_2_O_3_ and sewage water	400 W Newport Xenon lamp	IPCE = 2.3 and 3.6% at 390 nm H_2_ moles = 8.4 and 33.1 mmol·h^−1^·cm^−2^	[102]
SnO_2_:Ni,Ir Nanoparticulate photoelectrode	0.5 M HCl	400 W Newport Xenon lamp	J_ph_ = 46.38 mA/cm^2^ IPCE% = 17.43% at 307 nm H_2_ moles = 52.22 mmol·h^−1^·cm^−2^ at −1 V	[103]
Polyaniline/PbI_2_ nanocomposite	Sewage water	400 W Newport Xenon lamp	J_ph_= 0.077 mA.cm^−2^ at 390 nm H_2_ moles = 6 µmole·h^−1^·cm^−1^	[104]
Nanostructured Rock Wool (5 mg)	0.3 M Na_2_SO_4_ (pH = 7) aqueous solution	400 W Newport Xenon lamp	H_2_ moles = 1040.423 μmol/h·mg J_ph_ = 0.25 to 0.20 mA/mg IPCE = 9.77% @ 307 nm	This work

## Data Availability

Not applicable.

## Supplementary Materials

The following supporting information can be downloaded at https://www.mdpi.com/article/10.3390/nano12132169/s1, Figure S1: HR-TEM of the (A) RW_0.063_ and (B–D) RW_>0.3_ after hydrothermal technique at 140 °C and annealing; the inset of (D) shows SAED pattern of RW_>0.3_.
