# Supplementary material for: Fabrication and Characterization of Nanostructured Rock Wool as a Novel Material for Efficient Water-Splitting Application
Source: Nanomaterials (Basel). 2022 Jun 24;12(13):2169. doi: 10.3390/nano12132169 (PMC9267974; doi:10.3390/nano12132169)
Supplement: Supplementary file 1 [file nanomaterials-12-02169-s001.zip › nanomaterials-1751934-supplementary.pdf]

# Supplementary Data

## Fabrication and Characterization of Nanostructured Rock Wool as a Novel Material for Efficient Water-Splitting Application

**Sahar A. El-Gharbawy [1,2], Mawaheb Al-Dossari [3], Mohamed Zayed [4], Heba A. Saudi [1], Mohamed Y. Hassaan [1], Nada Alfryyan [5] and Mohamed Shaban [4,6,*]**

1. Department of Physics, Faculty of Science, Al-Azhar University (Girls' Branch), Nasr City, Cairo 11884, Egypt; fouads5649@gmail.com (S.A.E.-G.); heba_saudi@azhar.edu.eg (H.A.S.); myhassaan@yahoo.com (M.Y.H.)
2. Housing and Building National Research Center, 87 El-Tahrir St., Dokki, Giza 1770, Egypt
3. Department of Physics, Faculty of Science, King Khalid University, Abha 62529, Saudi Arabia; mdosri@kku.edu.sa
4. Nanophotonics and Applications (NPA) Lab, Department of Physics, Faculty of Science, Beni-Suef University, Beni-Suef 62514, Egypt; m.zayed88ph@yahoo.com
5. Department of Physics, College of Sciences, Princess Nourah Bint Abdulrahman University, P.O. Box 84428, Riyadh 11671, Saudi Arabia; naalfryyan@pnu.edu.sa
6. Physics Department, Faculty of Science, Islamic University of Madinah, P.O. Box 170, Al Madinah Al Monawara 42351, Saudi Arabia

* Correspondence: mssfadel@aucegypt.edu

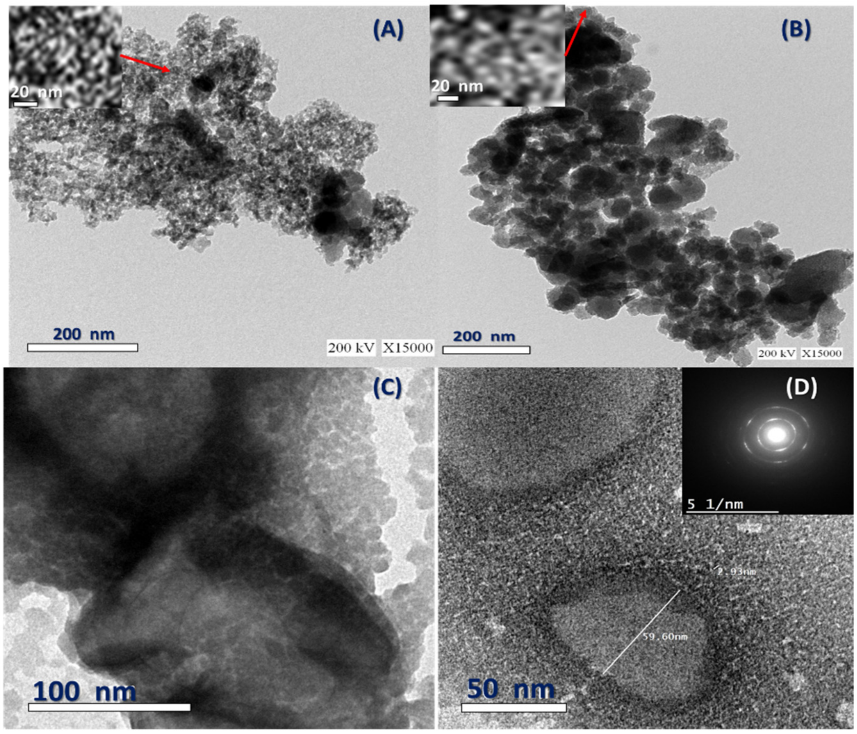


**Figure S1.** HR-TEM of the (**A**) $RW_{0.063}$ and (**B**–**D**) $RW_{>0.3}$ after hydrothermal technique at 140 °C and annealing; the inset of (**D**) shows SAED pattern of $RW_{>0.3}$.
